# Azolato-Bridged Dinuclear Platinum(II) Complexes Exhibit Androgen Receptor-Mediated Anti-Prostate Cancer Activity

**DOI:** 10.1021/acs.inorgchem.4c01093

**Published:** 2024-09-11

**Authors:** Tasuku Arai, Masashi Oshima, Masako Uemura, Takeshi Matsunaga, Taiki Ashizawa, Yoshitomo Suhara, Magotoshi Morii, Hiroki Yoneyama, Yoshihide Usami, Shinya Harusawa, Seiji Komeda, Yoshihisa Hirota

**Affiliations:** † Laboratory of Biochemistry, Department of Bioscience and Engineering, College of Systems Engineering and Science, Shibaura Institute of Technology, Saitama, Saitama 337-8570, Japan; ‡ Medicinal Chemistry and Organic Synthesis, Department of Systems Engineering and Science, Graduate School of Engineering and Science, Shibaura Institute of Technology, Saitama, Saitama 337-8570, Japan; § Department of Urology, Jichi Medical University Saitama Medical Center, Saitama, Saitama 330-8503, Japan; ∥ Division of Hematology and Oncology, Department of Internal Medicine, University of Cincinnati College of Medicine, Cincinnati, Ohio 45267, United States; ⊥ Faculty of Pharmaceutical Sciences, Suzuka University of Medical Science, Suzuka, Mie 513-8670, Japan; # Laboratory of Organic Synthesis and Medicinal Chemistry, Department of Bioscience and Engineering, College of Systems Engineering and Science, Shibaura Institute of Technology, Saitama, Saitama 337-8570, Japan; ∇ Department of Pharmaceutical Organic Chemistry, Faculty of Pharmacy, Osaka Medical and Pharmaceutical University, Takatsuki, Osaka 569-1094, Japan

## Abstract

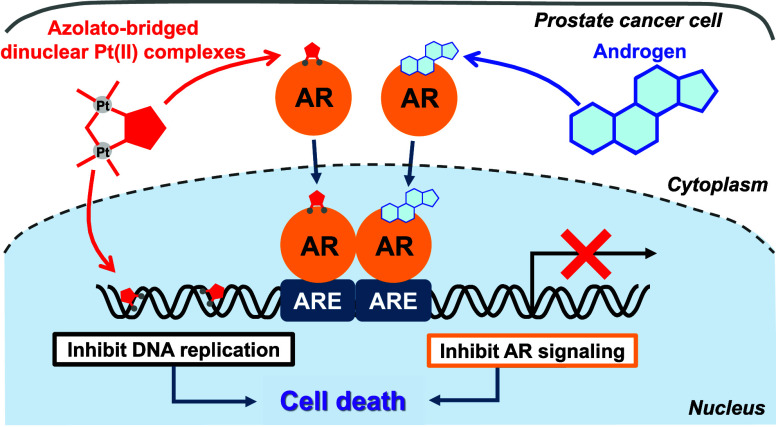

Prostate cancer is an androgen-dependent malignancy that presents a marked treatment challenge, particularly after progression to the castration-resistant stage. Traditional treatments such as androgen deprivation therapy often lead to resistance, necessitating novel therapeutic approaches. Previous studies have indicated that some of the azolato-bridged dinuclear platinum(II) complexes (general formula: [{*cis*-Pt(NH_3_)_2_}_2_(μ-OH)(μ-azolato)]X_2_, where azolato = pyrazolato, 1,2,3-triazolato, or tetrazolato and X = nitrate or perchlorate) inhibit androgen receptor (AR) signaling. Therefore, here we investigated the potential of 14 such complexes as agents for the treatment of prostate cancer by examining their antiproliferative activity in the human prostate adenocarcinoma cell line LNCaP. Several of the complexes, particularly **5-H-Y** ([{*cis*-Pt(NH_3_)_2_}_2_(μ-OH)(μ-tetrazolato-*N2*,*N3*)](ClO_4_)_2_), effectively inhibited LNCaP cell growth, even at low concentrations, by direct modulation of AR signaling, and by binding to DNA and inducing apoptosis, which is a common mechanism of action of Pt-based drugs such as cisplatin (*cis*-diamminedichloridoplatinum(II)). Comparative analysis with cisplatin revealed superior inhibitory effects of these complexes. Further investigation revealed that **5-H-Y** suppressed mRNA expression of genes downstream from AR and induced apoptosis, particularly in cells overexpressing AR, highlighting its potential as an AR antagonist. Thus, we provide here insights into the mechanisms underlying the antiproliferative effects of azolato-bridged complexes in prostate cancer.

## Abstract 2

Prostate cancer, especially at the castration-resistant stage, is challenging to treat. Traditional treatments often lead to resistance, hence the need for new methods. This study found that certain azolato-bridged dinuclear platinum(II) complexes inhibit androgen receptor signaling, which could be used in treatments. Testing on the LNCaP cell line showed several complexes, particularly **5-H-Y**, effectively inhibited cell growth by modulating AR signaling and inducing apoptosis. These complexes also outperformed cisplatin in inhibitory effects. Hence, azolato-bridged complexes could be a potential treatment for prostate cancer.

## Introduction

Prostate cancer is the second most commonly diagnosed cancer and the fifth leading cause of cancer-related deaths in men.^1^ In 2020, a total of 1,414,259 new cases of prostate cancer and 375,304 prostate cancer–related deaths were reported worldwide.^2^ Prostate cancer is an androgen-dependent cancer, and when localized treatments such as prostatectomy and radiotherapy fail, or in cases of primary metastatic cancer, a form of hormone therapy called androgen deprivation therapy targeting androgen signaling is indicated.^3,4^ Although androgen deprivation therapy is initially effective, some patients develop biochemical recurrence, as indicated by rising serum prostate-specific antigen (PSA) levels, eventually demonstrating clinical resistance, which manifests as the emergence of new metastases on computed tomography and other imaging modalities, as well as an increase in existing metastases. This is the most advanced stage of prostate cancer and is known as castration-resistant prostate cancer (CRPC).^5^ In CRPC, androgen receptor (AR), a transcription factor and nuclear receptor that is crucial for prostate development, is often overexpressed or hyperactivated.^6^ Therefore, treatments that target AR signaling, such as androgen receptor axis–targeted agents, taxane-based chemotherapies, and poly(ADP-ribose)polymerase inhibitors, are currently being developed; however, recent data indicate that these treatments show limited efficacy.^7^ Thus, since prostate cancer is difficult to treat once it progresses to CRPC, it has been proposed that potent agents such as taxanes^8^ and novel androgen receptor axis–targeted agents^9^ be introduced upfront during the hormone-sensitive phase of early CRPC.

**Figure 1:**
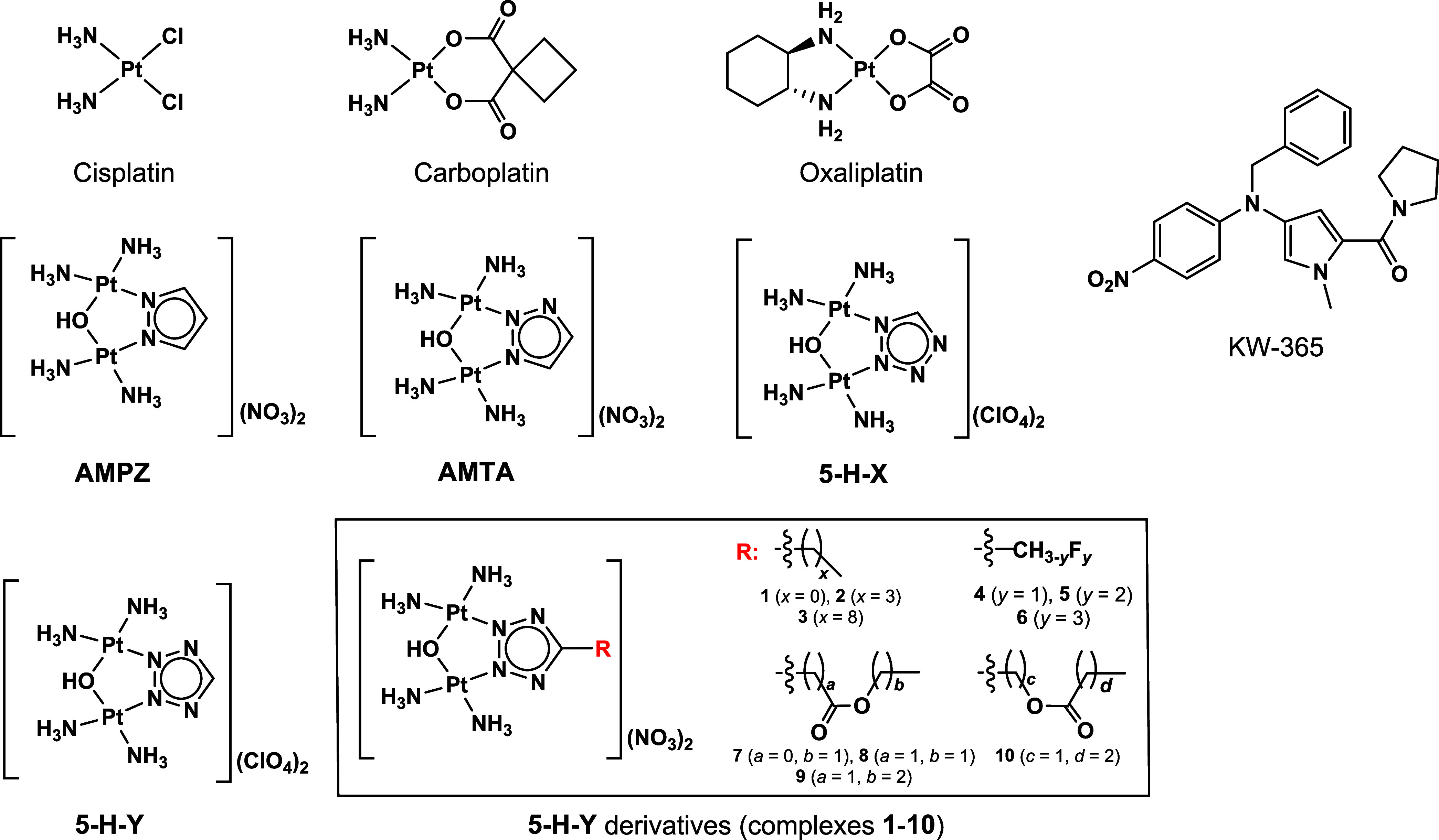
Molecular structures of three major Pt-drugs (cisplatin, carboplatin and oxaliplatin), a series of azolato-bridged dinuclear Pt(II) complexes (**AMPZ**, **AMTA**, **5-H-X**, **5-H-Y**), **5-H-Y** derivatives with the general formula [{*cis*-Pt(NH_3_)_2_}_2_(μ-OH)(μ-5-R-tetrazolato-*N2*,*N3*)](NO_3_)_2_ (complexes **1**–**10**), and the androgen antagonist KW-365.

Cisplatin, *cis*-diamminedichloridoplatinum(II), is an electrically neutral mononuclear Pt(II) complex that is currently used to treat various malignancies, including lung, ovarian, and bladder cancers, in which it induces high tumor reduction.^10^ Cisplatin and more recent related drugs such as carboplatin and oxaliplatin are collectively known as Pt-drugs (Figure 1) and together comprise one of the most widely used groups of anticancer drugs.^11^ However, the poor gastrointestinal absorption of cisplatin and its gastrointestinal stimulant effects mean that it must be administered by intravenous infusion, and poorly water-soluble cisplatin can accumulate in the kidneys, causing serious side effects such as acute renal failure.^12^ Considering that many CRPC patients already have compromised health, alternative treatments with fewer or less serious potential side effects, but potentially less efficacy, are often selected.^13^ A phase II clinical trial adding cisplatin to docetaxel, a cytotoxic anticancer drug that operates through the mechanism of inhibiting microtubule depolymerization for prostate cancer patients who have worsened postdocetaxel treatment, has revealed promising antitumor effects with a marked reduction of serum PSA in 48% of patients; however, due to severe side effects like renal and neurological damage, further clinical trials were halted.^14^

As a means of avoiding the toxicities associated with current treatment options, there is growing interest in developing Pt-drugs that are highly water soluble and recognize molecules specific to prostate cancer.^15^ Previously, Komeda et al. proposed a series of highly water-soluble azolato-bridged dinuclear Pt(II) complexes (azolato-bridged complexes) as a new class of Pt-drug candidates with the general formula [{*cis*-Pt(NH_3_)_2_}_2_(μ-OH)(μ-azolato)]X_2_ (azolato = pyrazolato, 1,2,3-triazolato, or tetrazolato; X = nitrate or perchlorate) (Figure 1), some of which exert high *in vivo* antitumor efficacy against pancreatic and colon cancers.^16−18^ The structure of azolato-bridged complexes is distinctive from that of existing Pt-drugs in that they possess two Pt(II) coordination planes and positive charges,^16,20−22^ which allows them to form unique Pt-DNA adducts.^21,23−25^ The formation of DNA adducts is thought to underly the efficacy of Pt-drugs.^26^ Importantly for the treatment of CRPC, a recent report has shown that two azolato-bridged complexes, **AMPZ** ([{*cis*-Pt(NH_3_)_2_}_2_(μ-OH)(μ-pyrazolato)](NO_3_)_2_) and **5-H-Y** ([{*cis*-Pt(NH_3_)_2_}_2_(μ-OH)(μ-tetrazolato-*N2*,*N3*)](ClO_4_)_2_), showed effective AR signal inhibition with half-maximal inhibitory concentrations of 5.4 and 10.1 μM (Komeda, unpublished data), respectively, in a screening assay^27^ using the AR-positive human prostate cancer cell line 22Rv1. This implies that the azolato-bridged complexes may act as antagonists that inhibit AR transcriptional activity. Given that existing AR antagonists, such as bicalutamide have been reported to bind with AR and be transported into the nucleus,^28^ it is hypothesized that the azolato-bridged complexes might follow a similar pathway, potentially offering therapeutic benefits.

Thus, it is of great interest to explore the potential of azolato-bridged complexes as therapeutic agents for the treatment of prostate cancer. In the present study, we evaluated the antiproliferative effects of 14 azolato-bridged complexes on a human prostate cancer cell line and analyzed the underlying molecular mechanisms, including the translocation process to the nuclear DNA. Particular focus is given to **5-H-Y**.

## Materials and Methods

### Chemicals

Azolato-bridged complexes and cisplatin were prepared as reported previously.^20,29^ The AR antagonist KW-365 was purchased from FUJIFILM Wako Pure Chemical Corporation (Tokyo, Japan). Dihydrotestosterone (DHT) was purchased from Tokyo Chemical Industry Corporation (Tokyo, Japan).

### Cell Culture and Reagents

The androgen-sensitive human prostate adenocarcinoma cell line LNCaP was obtained from RIKEN Bioresource Research Center (Ibaraki, Japan). LNCaP cells were cultured in Roswell Park Memorial Institute 1640 (RPMI-1640) medium (Nacalai Tesque, Kyoto, Japan) supplemented with 10% charcoal/dextran-stripped fetal bovine serum (FBS; Sigma-Aldrich, St. Louis, MO) and 50 U/mL penicillin and 50 μg/mL streptomycin (P/S) (Nacalai Tesque) at 37 °C under a humidified atmosphere containing 5% CO_2_. Cells were passaged upon reaching 80–90% confluence.

### Cell Viability Assay

Cell viability was determined with a water-soluble tetrazolium salt (WST-8) colorimetric assay using Cell Counting Kit-8 (Dojindo, Tokyo, Japan). LNCaP cells were seeded into 96-well plates (100 μL cell suspension/well) and cultured for 72 h with one or more of the test reagents, followed by the addition of 100 μL of freshly prepared medium containing 10 μL Cell Counting Kit-8. After incubation for 4 h at 37 °C under a humidified atmosphere containing 5% CO_2_, absorbance at 450 nm was measured using a microplate reader (Bio-Rad). Assays were carried out in quadruplicate, and data are presented as mean ± SD. Sigmoid curves were fitted to the data points using the KaleidaGraph analytical software version 4.5 (Synergy Software, Reading, PA) to calculate half maximal inhibitory concentrations (IC_50_) which were calculated as the concentration that provided 50% formazan production relative to the control (no Pt complex added).

### RNA Extraction and Quantitative Reverse Transcription Polymerase Chain Reaction (qRT-PCR) Analysis

Total RNA was isolated using Isogen reagent (Nippon Gene, Tokyo, Japan). Complementary DNA (cDNA) was synthesized using T100 Thermal Cycler (Bio-Rad, California) and ReverTra Ace qPCR RT Master Mix (Toyobo, Osaka, Japan). Amplification was performed using a CFX Connect Real-Time PCR Detection System (Bio-Rad) and THUNDERBIRD SYBR qPCR Mix (Toyobo). Fold changes for experimental groups relative to the loading control (β-actin) were calculated by means of the ΔCt method. The primer sequences were designed as follows:

β-actin forward: 5′-GGAGTCCTGTGGCATCCACGAAA-3′

β-actin reverse: 5′-CCACACGGAGTACTTGCGCTCAGG-3′

PSA forward: 5′-AGAGGAGTTCTTGACCCCAAAG-3′

PSA reverse: 5′-CTCCTTGGCGTTGTCAGAAATG-3′

TMPRSS forward: 5′-TAGTGAAACCAGTGTGTCTGCCCA-3′

TMPRSS reverse: 5′-AGCGTTCAGCACTTCTGAGGTCTT-3′

### Western Blot Analysis

LNCaP cells were harvested *via* trypsinization and washed twice with cold phosphate-buffered saline (PBS). Whole-cell lysates were prepared by three freeze–thaw cycles. Protein concentrations were measured by using a BCA Protein Assay Kit (Thermo Fisher Scientific, Waltham, MA). Protein was separated on 12% sodium dodecyl sulfate-polyacrylamide (SDS) gels, transferred to a poly(vinylidene fluoride) membrane (Bio-Rad), and blocked with Blocking-One solution (Nacalai Tesque). The blocked membrane was incubated with the primary antibodies anti-AR antibody (1:200, AR Clone 441; Dako, Copenhagen, Denmark) and anti-GAPDH antibody (1:5000, M171–7; Medical & Biological Laboratories, Tokyo, Japan) at 4 °C overnight. After being washed with TRIS-buffered saline (TBS) with 1% Triton X-100 (TBS-T), the membranes were incubated with horseradish peroxidase-conjugated antimouse secondary antibody (1:1000, sc-516102; Santa Cruz Biotechnology, Texas) at room temperature for 1 h. Blots were developed using enhanced Chemiluminescence ImmunoStar Zeta (Wako). Band intensities were densitometrically quantified by using the iBright CL1000 Imaging System (Thermo Fisher Scientific).

### Small Interfering RNA (siRNA) Transfection

LNCaP cells were transfected with control siRNA or siRNA targeting AR (siAR; 20 μM). Transfection was accomplished with Lipofectamine RNAi MAX reagent (Thermo Fisher Scientific) and cells were incubated for 24 h post-transfection. The control siRNA (sc-37007) and siAR (sc-29204) were purchased from Santa Cruz Biotechnology.

### AR Overexpression

#### Plasmid Transformation and Amplification

Competent *Escherichia coli* DH5α (Nippon Gene) were transformed with 5 μL of plasmid (1 μg/μL) using a heat shock method (42 °C, 45 s). Transformed cells were plated on PlusGrow II agar (Nacalai Tesque) and incubated at 37 °C in 5% CO_2_ for 16 h. Single colonies were selected and colony PCR was performed using KOD FX Neo (Toyobo) with specifically designed AR primers (forward: 5′-CCGCTGACCTTAAAGACATCCT-3′, reverse: 5′-CTCCTTGGCGTTGTCAGAAATG-3′). Amplification was confirmed on a 2% agarose gel.

#### Plasmid Purification

AR-positive colonies were cultured in PlusGrow II medium, followed by large-scale amplification. Plasmids were extracted using a NucleoBond Xtra Column kit (Takara Bio, Shiga, Japan). Concentration was determined with a DS-11 Spectrophotometer (DeNovix, Wilmington, DE) and adjusted to 1 μg/μL with TE buffer.

#### Cell Transfection

LNCaP cells were seeded in RPMI-1640 medium without phenol red or P/S and supplemented with 2.5% charcoal/dextran-stripped FBS. Cells were transfected with either pcDNA3.1 (GenScript, Piscataway, NJ) or pcDNA3.1-AR using PEI MAX (Polysciences, Warrington, PA) in Opti-MEM I Reduced Serum Medium (Thermo Fisher Scientific). After incubation for 24 h at 37 °C in 5% CO_2_, AR expression was assessed by qRT-PCR method.

### Immunofluorescence Staining

LNCaP Cells were seeded at 2.0 × 10^4^ cells/200 μL/well into poly-l-lysine-coated glass chambers and then incubated in phenol red-free RPMI-1640 medium enriched with 2.5% charcoal/dextran-stripped FBS for 72 h at 37 °C in 5% CO_2_. After incubation, the cells were rinsed 3 times with PBS, fixed using 100 μL of 4% paraformaldehyde (Wako) at room temperature for 20 min, permeabilized with 100 μL of 0.2% Triton-X (Wako) for 5 min at room temperature, and blocked with 100 μL of 1.5% skim milk for 1 h at room temperature. The primary AR antibody, which was diluted 200-fold in Can Get Signal Solution 1 (Toyobo), was applied and the cells were incubated at 4 °C overnight. After washing 3 times with PBS, the cells were treated with 400-fold diluted Alexa Fluor 594-conjugate Goat Anti-Mouse IgG (H + L) (Jackson ImmunoResearch Laboratories, West Grove, PA) in Can Get Signal Solution 2 (Toyobo) for 1 h at room temperature. Incubation with Hoechst 33342 solution (5 μg/mL final concentration) (Dojindo) for 30 min at room temperature was used for nuclear staining, and incubation with PlasMEM Bright Green solution (Dojindo), diluted 200-fold in PBS, for 20 min at 37 °C was used for membrane staining. The cells were visualized under a BZ-X800 fluorescence microscope (Keyence, Osaka, Japan) at the following excitation and emission wavelengths (excitation: AR 560 nm, nuclear 360 nm, membrane 470 nm; emission: AR 630nm, nuclear 460 nm, membrane 570 nm).

### Apoptosis Assay

LNCaP cells were seeded at 1.5 × 10^6^ cells/3 mL/well in phenol red-free RPMI-1640 medium, without P/S, and supplemented with charcoal/dextran-stripped FBS. After incubation for 24 h at 37 °C in 5% CO_2_, the test compound was added and the cells were incubated for another 24 h. For the apoptosis analysis, the cells were detached using Accutase (Nacalai Tesque), washed twice with PBS, and resuspended to a concentration of 1.0 × 10^6^ cells/mL in PBS. Then, 5 μM Annexin V-FITC (Nacalai Tesque) was added to 100 μL of the cell suspension and the mixture was incubated for 15 min in the dark at room temperature. Utilizing flow cytometry, the relative apoptotic frequency was determined in a sample of 1.0 × 10^4^ cells.

### Cell Cycle Assay

Cells were harvested and fixed with 70% ethanol at −30 °C overnight. Fixed cells were treated with RNase (Wako) and then stained with 50 μg/mL propidium iodide (Wako). The cells were analyzed by using a BD FACSAria III Cell Sorter (Becton Dickinson, Franklin Lakes, NJ). The proportions of cells in the sub-G1, G1, S, and G2/M phases of the cell cycle were evaluated. Each experiment was repeated 3 times, independently.

### Albumin Aggregation Assay

The samples were diluted in PBS to obtain 2 μg/mL Albumin concentration, and various platinum complexes were added to obtain 100 μM Cisplatin or **5-H-Y** concentrations, and then incubated for 2 h at 37 °C. A sample buffer containing 700 mM DTT was added to stop the chemical reaction between the Pt complexes and Albumin, and SDS-PAGE was performed. Post SDS-PAGE, the gel was immersed in Coomassie Brilliant Blue stain (CBB Stain One (Nacalai Tesque)) and incubated overnight. After incubation, the gel was removed from the stain and destained and dehydrated using 70% methanol. After dehydration, the gel was incubated again in the Coomassie Brilliant Blue staining solution for 1 h. The destaining process was iteratively repeated several times using 30% methanol.

### LNCaP Cell Suspension Aggregation Assay

LNCaP cell lysates were diluted with PBS containing a 10-fold concentration of serine protease inhibitors (Nacalai Tesque) to achieve a total protein concentration of 1.0 μg/μL. LNCaP cell lysates were treated with various compounds to obtain the specified final concentrations. The resulting samples were evaluated by Western blotting or Cell Thermal Shift Assay as described above.

### Quantification of Nuclear Pt Accumulation

LNCaP cells (2 × 10^5^ cells/mL) transfected with pcDNA3.1 or pcDNA3.1-AR were seeded in RPMI-1640 medium containing 2.5% charcoal-treated FBS. After 24 h, 1 μM Cisplatin or **5-H-Y** was added to the medium for 3 h, and then the cells were harvested. The recovered LNCaP cells were homogenized using a tissue homogenizer (Branson Ultrasonic). The homogenized cells were suspended in PBS and subjected to centrifugation at 900*g* for 10 min to isolate the nuclear fraction. The supernatant was discarded, and the nuclear pellet was further centrifuged using isosmotic sucrose solutions at concentrations of 0.26 M, 1.3 M, and 2.6 M to remove the mitochondrial fraction. The isolated nuclear fraction was washed in concentrated nitric acid by heating up to 120 °C. The dry residue was dissolved in 2 mL of 5%(v/v) HNO_3_. The amount of Pt was quantified by using a 7500CX inductively coupled plasma mass spectrometer (Agilent Technologies, Santa Clara, CA). Quantitative values were calculated and are shown in the figures in units of nanomoles of Pt complex per 10^7^ cells (Figure 6) because cisplatin contains one Pt(II) center, whereas **5-H-Y** contains two.

### Cell Thermal Shift Assay

LNCaP cells, cultured at 37 °C in 5% CO_2_ in RPMI-1640 medium supplemented with 10% FBS and 1% P/S (containing phenol red), were harvested at a concentration of 20 × 10^6^ cells/mL. After treatment with the test agents, the cells were incubated for 2 h at 37 °C, followed by thermal treatment at 37.0, 41.3, 45.7, 51.0, 55.1, and 60.0 °C for 3 min each. Protein lysates were prepared using a freeze–thaw method, and their concentration was determined with a BCA Protein Assay Kit. Samples were separated *via* SDS-PAGE, and AR proteins were labeled and detected using Western blotting targeting AR. Imaging of the AR protein bands was achieved with the iBright CL1000 Imaging System, and quantification was performed with the iBright Analysis Software Version 5.2.1 (Thermo Fisher Scientific). Sigmoid curves were created using the following equation: *y* = *a* + (*b* – *a*)/(1 + (*x*/*c*)^d^) on KaleidaGraph analytical software version 4.5 (Synergy Software). The temperature at which 50% of the protein was degraded was determined manually from the curve.

### Statistical Analyses

Statistical analyses were performed in Microsoft Excel 365 (Microsoft) or GraphPad Prism software version 9.0 (GraphPad Software, Inc.). In experiments using cell lines, data were compared using Student’s *t-*test to determine the statistical significance between groups. All data are expressed as mean ± SD unless otherwise indicated. *P* values < 0.05 were considered to be statistically significant. Other details are described in the figure legends.

## Results

### Azolato-Bridged Complexes Inhibit Prostate Cancer Cell Growth

**Figure 2:**
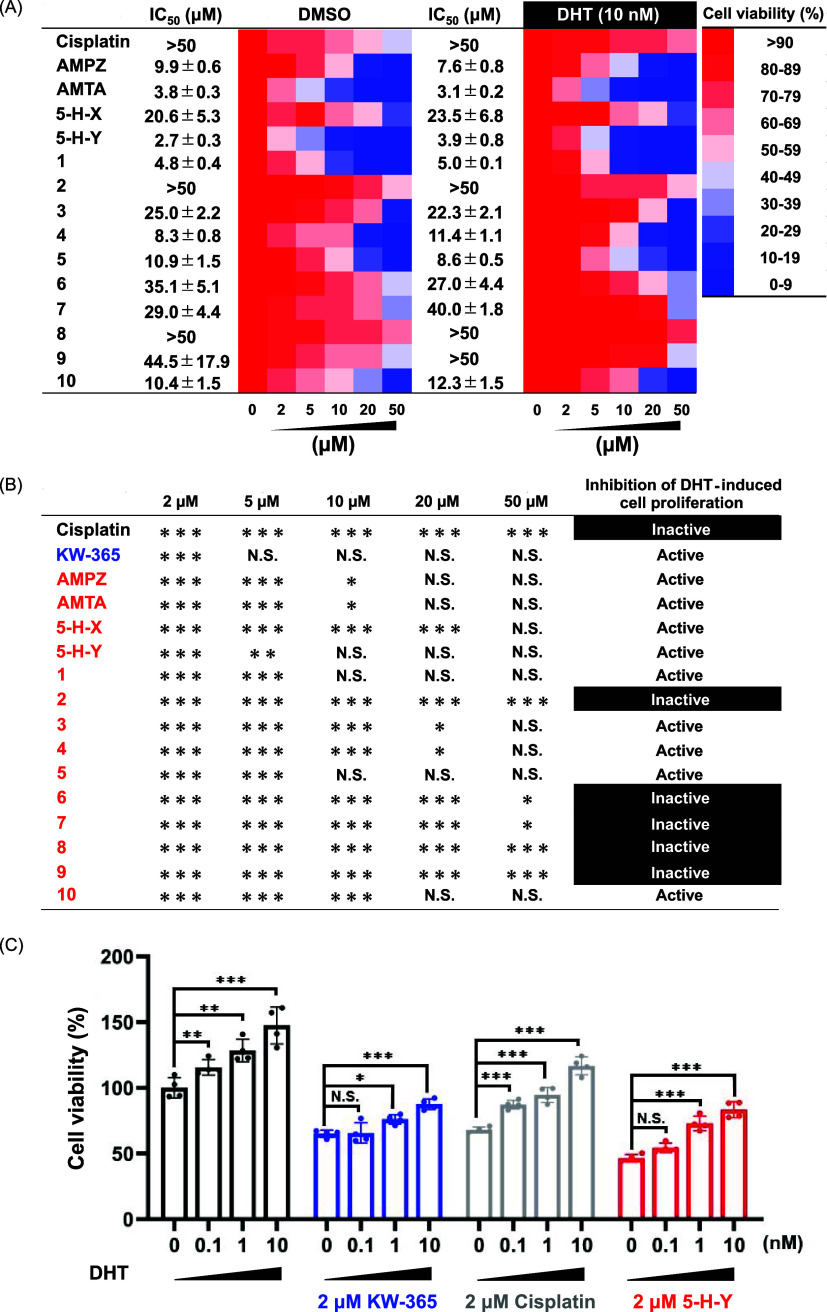
Inhibition of prostate cancer cell proliferation by 14 azolato-bridged dinuclear Pt(II) complexes. Cells of the androgen receptor (AR)–positive prostate cancer cell line LNCaP were treated with the indicated azolato-bridged complexes in the presence or absence of the androgen dihydrotestosterone (DHT). (A) Concentration-dependent inhibition of cancer cell proliferation by Pt complexes on LNCaP cells in the presence of 10 nM DHT. Dimethyl sulfoxide (DMSO) was used as a control, and the DMSO content in any medium was kept below 0.1%. (B) Analysis of the inhibitory effect of DHT on cell proliferation of LNCaP cells: comparison of the proliferative effect of 10 nM DHT-treated cells ((A) right) against cells not treated with DHT ((A) left). (C) Effect of KW-365 and cisplatin, **5-H-Y** on DHT concentration-dependent cell viability. The structures of the compounds are shown in Figure 1. *p < 0.05, **p < 0.01, ***p < 0.001, N.S.; not significant compared with vehicle.

We began by examining the effects of cisplatin and of 14 azolato-bridged complexes (0–50 μM) on the viability of LNCaP cells, an AR-positive prostate cancer cell line (Figure 2A and Supporting Figure 1). Treatment with each of the complexes alone (vehicle, dimethyl sulfoxide) decreased cell viability in a dose-dependent manner. Similarly, when the cells were treated with each of the complexes in the presence of the androgen dihydrotestosterone (10 nM; DHT), which induces proliferation of LNCaP cells, cell viability was decreased in a dose-dependent manner. Furthermore, all azolato-bridged complexes, except for complexes **2**, **8** and **9**, showed lower half-maximal inhibitory values compared with cisplatin. The structure-activity relationships were similar to those previously reported^30^ for complexes **1**–**3**^31^ and **4**–**6**.^18^ The *in vitro* cytotoxicity of complexes **1**–**3** with linear alkyl chains at tetrazole C5 was found to have a U-shaped association with alkyl chain length. That is, medium-length alkyl substituents exhibited the least cytotoxicity. For complexes **4**–**6**, the *in vitro* cytotoxicity of the complexes decreased with the increasing number of fluorine atoms, introduction of which into a lead compound is a common means of modulating the polarity or stability of the compound without greatly increasing its molecular size, and the cytotoxicity tendency was found to be similar to that reported for colon-26 mouse colorectal cancer cells.^18^

Next, based on these findings, we further explored the inhibitory effects of the azolato-bridged complexes on DHT-induced cell proliferation in LNCaP cells (Figure 2B and Supporting Figure 1). Cisplatin exhibited weak inhibitory effect on DHT-induced cell proliferation, while the AR antagonist KW-365 (positive control) showed a strong inhibitory effect. Among the azolato-bridged complexes, **AMPZ**, **AMTA**, **5-H-X**, **5-H-Y**, and complexes **1**, **3**–**5**, and **10** displayed low half-maximal inhibitory concentrations for LNCaP cell growth in the absence of DHT (Figure 2A). Although the reactivity of azolato-bridged complexes toward nucleophiles is largely unchanged by the introduction of substituents,^32^*in vitro* activity can vary greatly depending on the type of substituent.^30^ In addition, the formation of noncovalent adducts with target biomolecules and intracellular accumulation of the complexes may be affected.^18,20,25,31,33^ In the present study, the azolato-bridged complexes with no substituents on the azolate ring (*i.e.*, **AMPZ**, **AMTA**, and **5-H-Y**) showed high antiproliferative activity against LNCap cells. The reactivity of these compounds increased as the number of nitrogen atoms in the bridging azolate ring increased; therefore, their reactivity increased in the order **AMPZ** < **AMTA** < **5-H-Y**.^32,34^ However, their *in vitro* antiproliferative activities were comparable. Although the relationship between reactivity and *in vitro* activity is uncertain, the *in vivo* acute toxicity of these three complexes was lower for the more reactive compounds (Komeda, unpublished data). Therefore, it was considered appropriate to select a drug candidate compound from the series of tetrazolato-bridged complexes, among which **5-H-Y**, and complexes **1** and **5** significantly inhibited DHT-induced proliferation of LNCaP cells. Consequently, **5-H-Y**, with the second lowest half-maximal inhibitory concentration for DHT-induced proliferation of LNCaP cells (Figure 2B), was selected for further investigation. In fact, **5-H-Y** is already considered a promising anticancer drug candidate due to its high *in vivo* antitumor efficacy against pancreatic cancer.^16^**AMPZ**, **AMTA** (Komeda, unpublished data) and **1**(18) were excluded due to their tendency to exhibit acute toxicity when administered to mice. In this study, **5-H-Y** not only robustly inhibited DHT-dependent cell proliferation but also reduced LNCaP cell viability even at low concentrations, suggesting that **5-H-Y** could be a potential candidate for the development of anticancer drugs for the treatment of prostate cancer.

We next used a cell viability assay to compare the inhibitory effects of cisplatin, KW-365, and **5-H-Y** on DHT-induced cell viability (Figure 2C). In the presence of DHT alone (0–10 nM), LNCaP cells exhibited growth that was proportional to DHT concentration. Consistent with our earlier finding (Figure 2B), treatment with 2 μM cisplatin did not significantly inhibit DHT dose-induced viability. In contrast, treatment with 2 μM KW-365 or **5-H-Y** did significantly inhibit DHT-induced cell viability at low (0 or 0.1 nM), but not high (1 or 10 nM), DHT concentrations.

### **5-H-Y** Modulates Prostate Cancer Cell Growth and Apoptosis through AR Signaling

**Figure 3:**
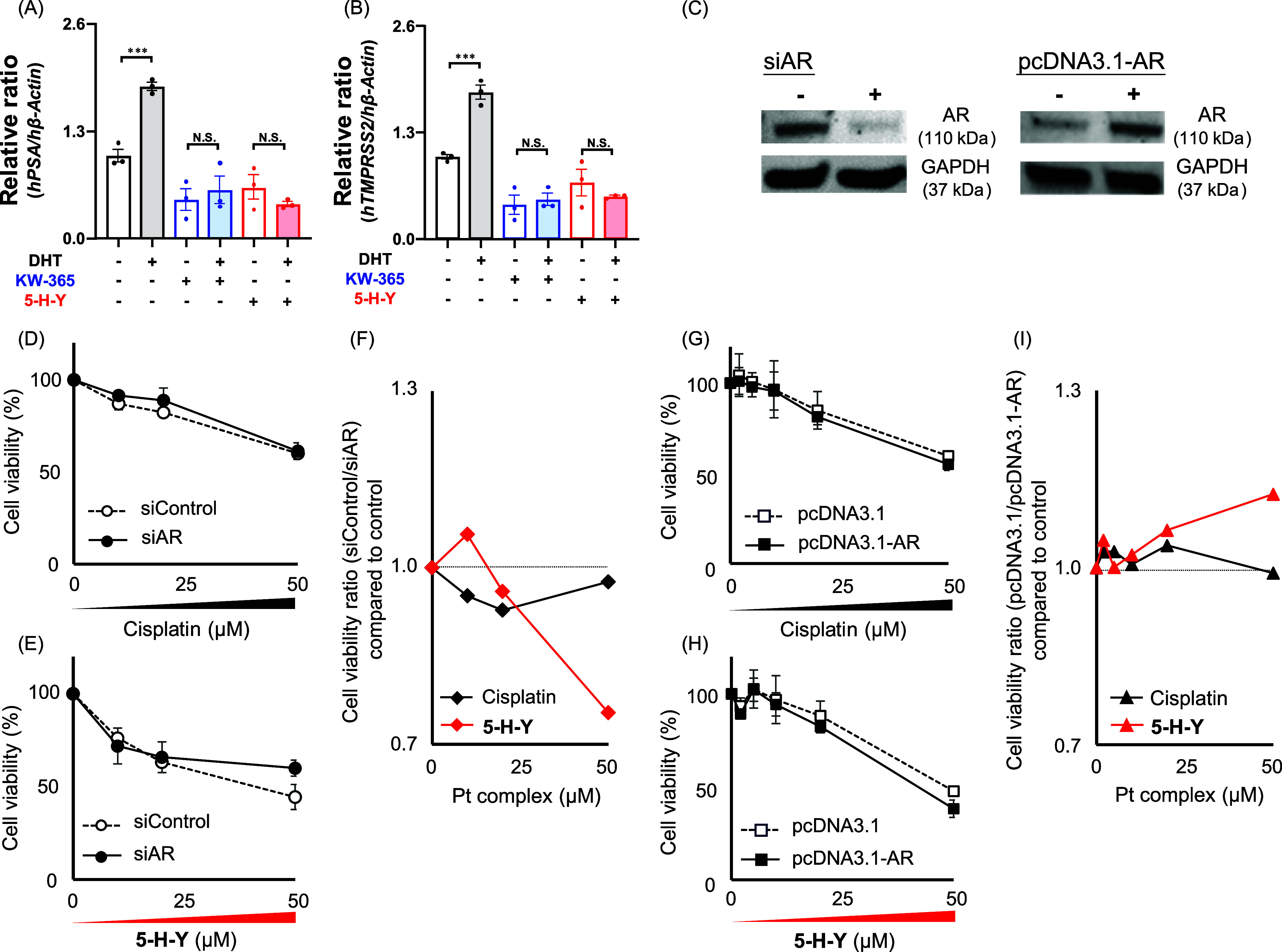
**5-H-Y** suppresses androgen receptor (AR) signaling and inhibits prostate cancer cell proliferation. (A, B) RNA expression levels of the AR-responsive genes encoding prostate-specific antigen (PSA) and transmembrane protease, serine 2 (TMPRSS2) in LNCaP cells after treatment with **5-H-Y**. Conditions: dihydrotestosterone (DHT), 0.13 nM; KW365, 2 μM; **5-H-Y**, 50 μM. ***p < 0.001, N.S.; not significant. (C) Western blots confirming that the small interfering RNA targeting AR (siAR) decreased endogenous AR expression, and that the AR expression plasmid (pcDNA3.1-AR) increased endogenous AR expression, in LNCaP cells. (D–F) Effects of siAR-induced knockdown of AR expression on the inhibition of cell viability by cisplatin and **5-H-Y** in LNCaP cells. (G–I) Effects of pcDNA3.1-AR-induced overexpression of AR on the inhibition of cell viability by cisplatin and **5-H-Y** in LNCaP cells.

To investigate the effect of **5-H-Y** on prostate cancer cell proliferation *via* AR signaling, we evaluated the mRNA expression levels of the AR-responsive genes encoding PSA and transmembrane protease, serine 2 (TMPRSS2) after **5-H-Y** treatment in LNCaP cells (Figure 3A,B). Pretreatment with DHT induced a significant increase in the mRNA expression of PSA and TMPRSS2 compared with no pretreatment. However, pretreatment with KW-365 or **5-H-Y** negated any DHT-induced increase in both mRNA expressions.

Next, we used an siRNA targeting AR (siAR) to knock down the expression of AR in LNCaP cells. Western blot analysis confirmed a substantial reduction in endogenous AR expression in LNCaP cells treated with siAR compared with those treated with a control siRNA (Figure 3C, left). In AR-knockdown LNCaP cells, cisplatin showed no inhibitory effect on cell viability (Figure 3D,F), whereas 50 μM **5-H-Y** showed an increased inhibitory effect (Figure 3E,F).

We then used an AR expression plasmid (pcDNA3.1-AR) to induce overexpression of AR in the LNCaP cells and again examined the inhibitory effects of cisplatin and **5-H-Y** on cell viability (Figure 3G–I). Before the experiment, we confirmed by Western blot that the pcDNA3.1-AR expression plasmid increased endogenous AR expression (Figure 3C, right). In cells overexpressing AR, the inhibitory effect of cisplatin on cell viability remained unchanged (Figure 3G,I), whereas that of 50 μM **5-H-Y** was increased (Figure 3H,I).

**Figure 4:**
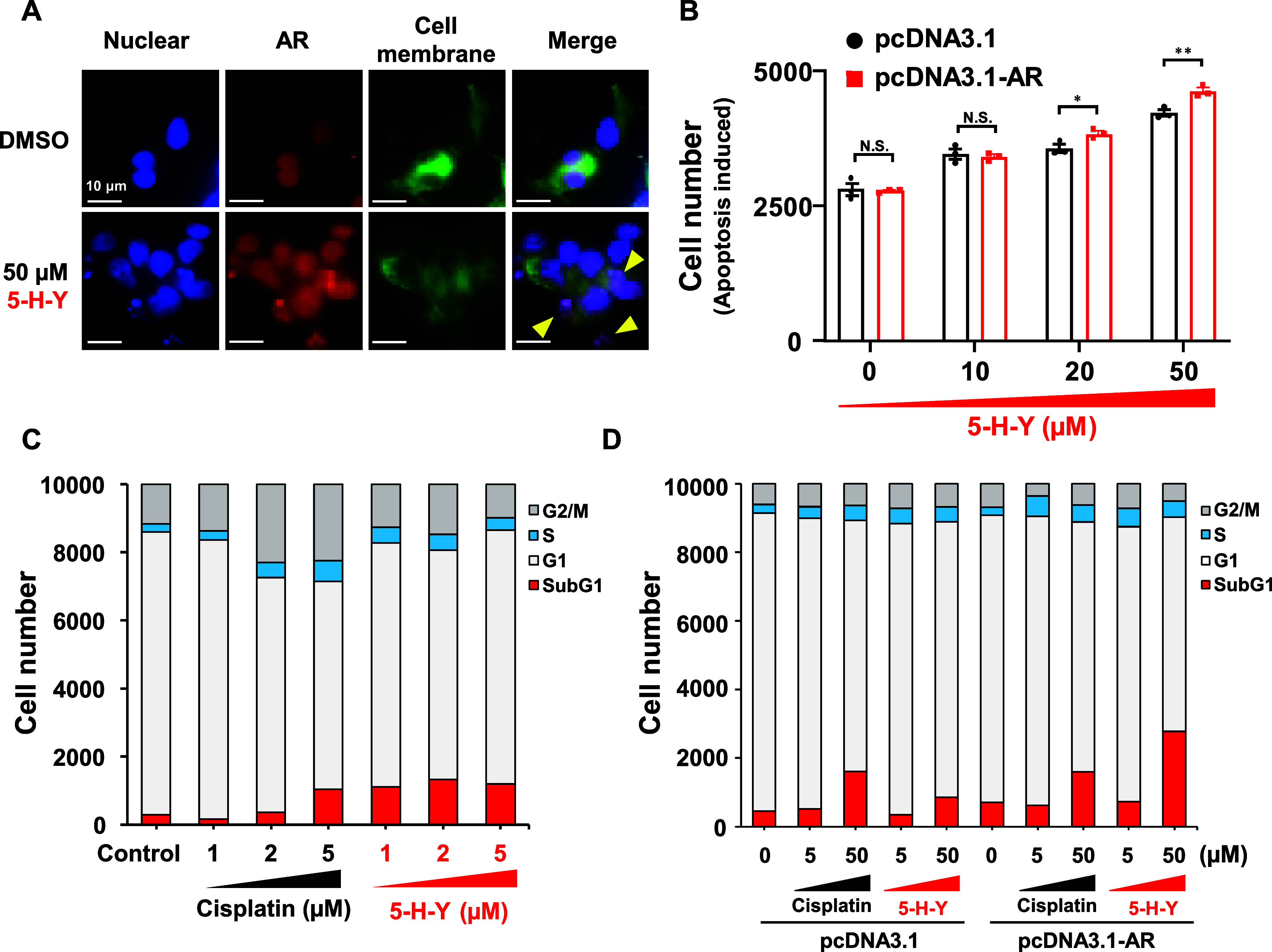
**5-H-Y** induces apoptosis in prostate cancer cells through androgen receptor (AR) signaling. (A) Immunofluorescence images showing the induction of apoptosis in LNCaP cells treated with **5-H-Y**. Arrows indicate areas where apoptosis occurred. DMSO, dimethyl sulfoxide. (B) Effect of concentration on the degree of apoptosis induced by **5-H-Y** in LNCaP cells overexpressing AR (pcDNA3.1-AR) or not (pcDNA3.1). *p < 0.05, **p < 0.01, N.S.; not significant. (C) Effects of cisplatin and **5-H-Y** on cell cycle in LNCaP cells. (D) Effects of cisplatin and **5-H-Y** on cell cycle in LNCaP cells overexpressing AR or not.

We next used immunofluorescence staining and flow cytometry to evaluate the ability of **5-H-Y** to induce apoptosis in LNCaP cells (Figure 4). The immunofluorescence analysis revealed that treatment with **5-H-Y** induced apoptosis in the LNCaP cells, resulting in fragmentation of DNA (Figure 4A). Staining for AR revealed subcellular localization of AR in the fragmented chromatin aggregates coincided with the fragmentation of chromatin aggregates. Furthermore, the apoptosis-inducing effect of a high concentration of **5-H-Y** (20 or 50 μM) was significantly stronger in cells overexpressing AR than in cells with normal expression (Figure 4B). In pcDNA3.1-treated cells, the percentage of dead cells to the total number of cells at each concentration of **5-H-Y** was 4.7% at 0 μM, 5.2% at 10 μM, 5.5% at 20 μM, and 6.8% at 50 μM. In pcDNA3.1-AR–treated cells, the percentage of dead cells to the total number of cells was 4.9% at 0 μM, 5.2% at 10 μM, 5.9% at 20 μM, and 7.6% at 50 μM of **5-H-Y**. The percentage of dead cells was increased significantly with 20 μM and 50 μM of **5-H-Y** (Figure 4B).

To understand more about how **5-H-Y** induced apoptosis, we compared the effects of treatment with 1–5 μM cisplatin or **5-H-Y** on the LNCaP cell cycle (Figure 4C). Cisplatin and **5-H-Y** both induced apoptosis by inhibiting the transition of LNCaP cells to the G2/M and G1 phases in a concentration-dependent manner, although **5-H-Y** induced apoptosis of LNCaP cells with higher efficiency than did cisplatin at both concentrations. In addition, **5-H-Y** induced apoptosis of LNCaP cells more efficiently than did cisplatin at all concentrations examined. These findings are consistent with previous reports showing that two other cancer cell lines, Hela (human cervical cancer) and PC9 (human nonsmall-cell lung cancer) cells, exposed to 2 μM **5-H-Y** were arrested in the S/G2 phase.^35^ In LNCaP cells overexpressing AR, no effects on the cell cycle were observed with cisplatin treatment (0–50 μM). However, **5-H-Y** treatment (0–50 μM) strongly suppressed transition to the G2/M phase and increased the number of cells in the sub-G1 phase in cells overexpressing AR. Apoptosis was induced most efficiently in **5-H-Y**-treated cells overexpressing AR (Figure 4D).

### **5-H-Y** Binds to and Antagonizes AR

**Figure 5:**
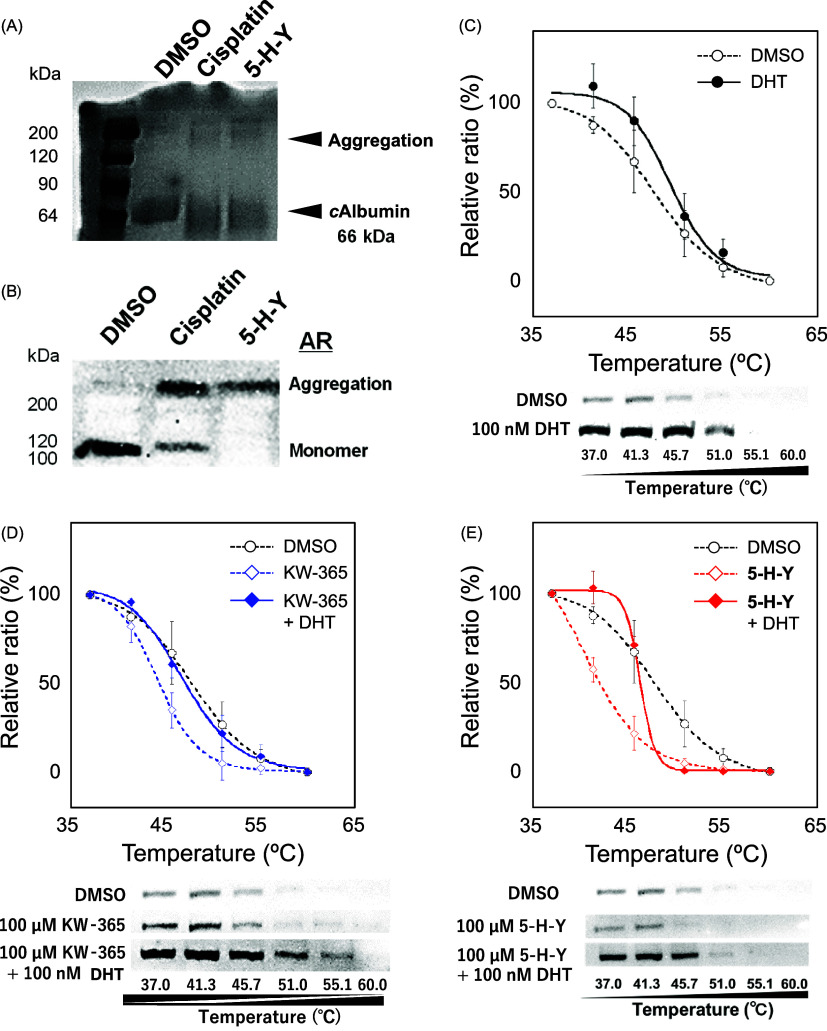
**5-H-Y** binds directly to androgen receptor (AR). (A) Coomassie Brilliant Blue stain confirming that bovine serum albumin forms aggregates in the presence of **5-H-Y**. (B) Western blot confirming that AR forms aggregates in the presence of **5-H-Y** or cisplatin. (C–E) Cellular thermal shift assay results for AR after treatment with dihydrotestosterone (DHT), KW-365, or **5-H-Y**. DMSO, dimethyl sulfoxide (vehicle).

The compounds in the series of azolato-bridged complexes represented by **5-H-Y** each have two positive charges and an OH bridge that is relatively stable compared to the chloride ligands of cisplatin that together act as a leaving group for covalent DNA binding.^32,34^**5-H-Y** binds to DNA through an interaction involving two different steps that may be characterized as noncovalent and covalent DNA binding, respectively, with the phosphate backbone^25^ and with guanine base.^36^ The former interaction is characteristic of azolato-bridged complexes and is not observed in electrically neutral Pt-drugs, whereas the latter is the common binding site for both azolato-bridged complexes and Pt-drugs. Indeed, the multimodal DNA binding of **5-H-Y** leads to the induction of highly efficient compaction of T4 phage DNA (166 kbp)^31,33,37^ and nucleosome.^35^ When interacting with proteins such as human serum albumin or AR-ligand binding domain (AR-LBD), *in silico* experiments using AutoDock have predicted that **5-H-Y** noncovalently binds to acidic amino acid clusters through electrostatic interactions and multiple hydrogen bonds between the ammine ligands of **5-H-Y** and the carboxylate ions of glutamate and aspartate residues (M. Morii, unpublished data). Such a noncovalent interaction may induce conformational changes in AR-LBD, which contains 11 glutamate and 11 aspartate residues, as well as 13 methionine and 6 cysteine residues, and the number of cysteine thiol groups that remain as thiol groups is theoretically four. On the other hand, it has also been confirmed that the OH bridge of azolato-bridged complexes can be readily replaced by the thiol group of glutathione at room temperature, as assessed by nuclear magnetic resonance spectroscopy (M. Uemura, unpublished data). Thus, as with DNA compaction, noncovalent and covalent Pt-protein adducts are likely to be formed on proteins. The former is reversible and characteristic of **5-H-Y**, but the latter is not reversible and common for **5-H-Y** and Pt drugs such as cisplatin. Anticancer Pt(II) complexes are known to interact with proteins through cysteine, methionine, and histidine,^38^ leading to the formation of protein aggregates. Cysteine and methionine, which both contain sulfur, are particularly reactive amino acid residues with Pt(II) complexes. We investigated the binding of cisplatin and **5-H-Y** to bovine serum albumin, a protein known for its affinity with cisplatin.^39^ Coomassie Brilliant Blue staining analysis confirmed that both cisplatin and **5-H-Y** induce aggregation of bovine serum albumin (Figure 5A). In a similar manner, we assessed the aggregation between AR and cisplatin or **5-H-Y** using LNCaP cell suspensions and found that both compounds formed aggregates with AR (Figure 5B).

We then assessed the effects of DHT, KW-365, and **5-H-Y** on the thermal stability of AR by using the cellular thermal shift assay. Dimethyl sulfoxide-treated AR showed temperature-dependent degradation, with 50% of the protein at 37 °C degraded at 47.9 °C. In contrast, the thermal stability of AR was increased with DHT treatment such that 50% of the protein at 37 °C was not degraded until 50.0 °C (Figure 5C). KW-365 treatment decreased the thermal stability of AR compared with that with dimethyl sulfoxide treatment, with 50% protein degradation occurring at 44.4 °C (Figure 5D). Combination treatment with DHT + KW-365 decreased the thermostability of AR compared with that with DHT treatment alone, with 50% degradation occurring at 47.2 °C. The same trends were observed for **5-H-Y**: with **5-H-Y** treatment alone, 50% protein degradation occurred at 42.1 °C, and with combination DHT + **5-H-Y** treatment protein degradation occurred at 46.5 °C (Figure 5E). Together, these findings indicate that **5-H-Y** may act as an AR antagonist like KW-365.

### **5-H-Y** Binds to AR and Is Transported to the Nucleus

**Figure 6:**
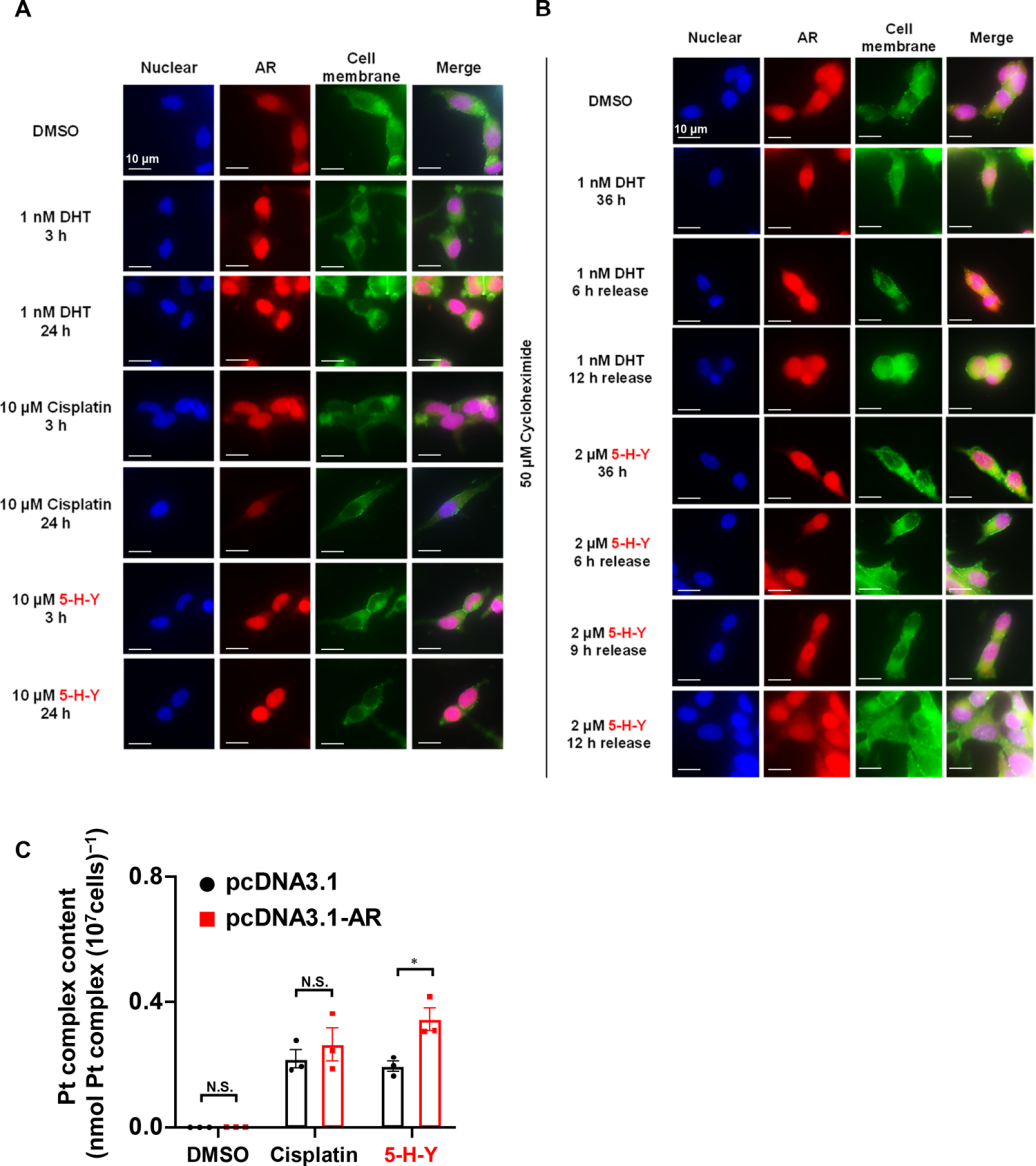
Androgen receptor (AR) binds to **5-H-Y** and is transported to the nucleus. (A) Fluorescence immunostaining showing the time-dependent changes in AR localization following treatment with dihydrotestosterone (DHT), cisplatin, or **5-H-Y**. (B) Fluorescence immunostaining showing the time-dependent nuclear export of AR at 36 h after **5-H-Y** treatment. (C) Changes in nuclear Pt accumulation following administration of cisplatin or **5-H-Y** under conditions of AR overexpression. *p < 0.05, N.S.; not significant. DMSO, dimethyl sulfoxide (vehicle).

To investigate the potential AR-mediated nuclear uptake of azolato-bridged complexes, LNCaP cells were treated with DHT, cisplatin, or **5-H-Y**. Subsequent fluorescence immunostaining allowed us to observe AR localization (Figure 6A). DHT-treated cells exhibited increased accumulation of AR in the nucleus over time compared to dimethyl sulfoxide-treated cells. In contrast, cisplatin-treated cells did not display any significant nuclear accumulation of AR over the same duration. Similar to those treated with DHT, cells treated with **5-H-Y** showed a time-dependent nuclear accumulation of AR.

Next, we assessed the changes in AR localization in LNCaP cells after 36 h treatment with DHT, cisplatin, or **5-H-Y** followed by a switch to a medium without the test compound (Figure 6B). AR was initially localized in the nucleus by the DHT or **5-H-Y** treatment, but then transitioned into the cytoplasm in a time-dependent manner upon the switch to the ligand-free medium. To confirm the observed AR-mediated nuclear accumulation of cisplatin and **5-H-Y**, we quantitatively assessed the accumulation of Pt in the nuclear fraction of AR-overexpressing LNCaP cells treated with cisplatin or **5-H-Y** (Figure 6C). **5-H-Y** treatment significantly increased the concentration of Pt in the nuclear fraction of AR-overexpressing LNCaP cells, whereas cisplatin treatment did not.

## Discussion

Azolato-bridged complexes are some of the most promising next-generation Pt-drug candidates. They are known to exert their anticancer activity by inhibiting DNA replication^35^ and gene expression^40^*via* unique multimodal DNA bindings^18,24,25,31,33,35,37,41^ that lead to apoptosis of cancer cells. DNA is a common target for most anticancer Pt complexes, meaning it would be of great interest if any of the azolato-bridged complexes had a unique target molecule other than DNA, and if that molecule was characteristic of one or more particular cancers, it would help determine which cancers are potentially susceptible to the cytotoxic effects of the azolato-bridged complex.

In the present study, we examined the inhibitory effects of 14 azolato-bridged complexes on the proliferation of a human prostate cancer cell (LNCaP), with a particular emphasis on the azolato-bridged complex **5-H-Y**. This Pt(II) complex distinguished itself through its potent efficacy even at low concentrations (Figure 2A). Our findings indicate that **5-H-Y** binds directly to AR then translocates to the nucleus, that it suppresses cell proliferation by suppressing AR signaling, and that it induces apoptosis by binding to DNA. Although the DNA interaction modes are different between Pt-drugs and azolato-bridged complexes,^23,24,42^ they both inhibit DNA replication and trigger cell death by forming covalent bonds with DNA.^35^ To the best of our knowledge, this is the first report demonstrating that a Pt(II) complex without any androgen derivative in the ligand inhibits prostate cancer growth through AR signaling.

Our comparative analysis of the inhibitory effects of the azolato-bridged complexes on the proliferation of AR-positive prostate cancer cell line LNCaP, relative to the established Pt-drug cisplatin, revealed nine compounds that demonstrated superior inhibitory effects (Figure 2A). Intracellular accumulation of Pt is considered critical for the anticancer effect of Pt(II) complexes, and it has been shown that azolato-bridged complexes are taken up more efficiently into cancer cells than is cisplatin.^20^ In a previous study examining the intracellular accumulation of **5-H-Y** and complexes **1** and **2** in a nonsmall-cell lung cancer cell line (NCI-H460),^31^**5-H-Y** showed higher intracellular accumulation than the other two complexes, suggesting a possible link to their differing abilities to inhibit DHT-induced LNCaP cell proliferation.

Prostate cancer is an androgen-dependent tumor, and AR signaling is crucial for its growth and progression.^3^ AR localized in the cytoplasm is normally bound to heat shock proteins, such as HSP70 and HSP90. Upon binding of ligands such as DHT, AR undergoes structural changes, dissociates from the heat shock proteins, and becomes activated.^43^ The active form of AR then moves from the cytoplasm to the nucleus, where it forms dimers that then form complexes with coactivators to bind to genomic sequences known as androgen response elements. Androgen response elements are located in the promoter or enhancer regions of target genes and promote their transcription.^44^ In addition to its transcriptional activation role through binding to androgen response elements, AR also regulates various gene expressions through interactions with transcription factors such as forkhead box A1, GATA binding protein 2, homeobox protein 13, and octamer transcription factor 1.^45^ AR antagonists like bicartamide and flutamide, frequently used in clinical hormone therapy for prostate cancer, act as inhibitory ligands, reducing DHT binding frequency, thereby suppressing AR signaling and inhibiting prostate cancer cell proliferation.^46^ Our findings extend this understanding by showing that **5-H-Y**, similar to AR antagonists, suppresses LNCaP cell proliferation induced by low concentrations of DHT. High DHT concentrations, however, did not inhibit cell proliferation, suggesting competitive binding of **5-H-Y** to the AR ligand-binding domain. Furthermore, **5-H-Y** suppressed DHT-induced mRNA expression of two genes encoding proteins downstream from AR (PSA and TMPRRS2) similar to the activity of the AR antagonist KW-365. Indeed, the inhibitory effect of **5-H-Y** on LNCaP cell proliferation decreased with AR expression suppression and increased with AR overexpression, whereas the activity of cisplatin was unaffected by these changes in AR expression levels. Also, **5-H-Y** induced apoptosis in LNCaP cells, and this apoptotic effect was stronger in AR-overexpressing cells, suggesting that AR is involved in **5-H-Y**-induced apoptosis, and that the antiproliferative effect of **5-H-Y** in LNCaP cells through AR involves cell death *via* apoptosis.

The present study also provides insights into the specific interaction between AR and **5-H-Y**. Cell thermal shift assays revealed that **5-H-Y** directly binds to AR and acts as an AR antagonist. In contrast, cisplatin, despite forming aggregates with AR, did not show a proportional inhibitory effect on prostate cancer cell proliferation in accordance with AR expression level. This difference highlights the unique properties of azolato-bridged complexes in potentially serving as selective therapeutic agents for prostate cancer.

Finally, the nuclear and cytoplasmic transport pathways of **5-H-Y** were also examined. In LNCaP cells treated with **5-H-Y**, AR was transferred to the nucleus in a time-dependent manner, similar to DHT, and, furthermore, AR was transferred to the nucleus with **5-H-Y** and remained in the cytoplasm even after **5-H-Y** was removed. This suggests that the azolato-bridged complexes are transported by AR to the nucleus, where they could be partially released. Indeed, AR is known to have a ligand-regulated nuclear export signal^47^ and **5-H-Y** treatment resulted in the accumulation of Pt in the nuclear fraction of LNCaP cells, with this tendency being more pronounced in AR-overexpressing cells. In contrast, this was not observed with cisplatin.

Thus, the present findings confirm that **5-H-Y** selectively inhibits proliferation in human prostate cancer cells *via* a mechanism that involves translocation to the nucleus, either directly or *via* AR binding, followed by induction of apoptosis.

## Conclusions

Pt-drugs are believed to initiate anticancer activity by binding to DNA, but they also bind to other biomolecules, meaning that they do not necessarily recognize cancer-type-specific biomolecules. Although the azolato-bridged complexes also exert anticancer activity *via* a mechanism that is similar to that of current Pt-drugs, they also appear to interact with AR, which is a crucial molecule for the proliferation of prostate cancer cells. The present results indicate that one of the azolato-bridged complexes, **5-H-Y**, effectively inhibits prostate cancer cell proliferation by binding to AR, as well as by inducing apoptosis after DNA-binding. Our findings underscore the potential of **5-H-Y**, and the azolato-bridged complexes as a group, to be used as novel chemotherapeutic agents for the treatment of prostate cancer.

## Supporting Information Available

Cell viability profiles for all compounds (PDF)

## Abbreviations

AR: androgen receptor
CRPC: castration-resistant prostate cancer
DHT: dihydrotestosterone
