# Supplementary material for: Azolato-Bridged Dinuclear Platinum(II) Complexes Exhibit Androgen Receptor-Mediated Anti-Prostate Cancer Activity
Source: Inorg Chem. 2024 Sep 11;63(44):20951–63. doi: 10.1021/acs.inorgchem.4c01093 (PMC11539055; doi:10.1021/acs.inorgchem.4c01093)
Supplement: Supplementary file 1 — ic4c01093_si_001.pdf [file ic4c01093_si_001.pdf]

Supporting Information

Azolato-bridged dinuclear platinum(II) complexes exhibit androgen receptor–mediated anti–prostate cancer activity

Tasuku Arai[1,2,+], Masashi Oshima[1,3,4,+], Masako Uemura[5], Takeshi Matsunaga[1], Taiki Ashizawa[1,2], Yoshitomo Suhara[2,6], Magotoshi Morii[5], Hiroki Yoneyama[7], Yoshihide Usami[7], Shinya Harusawa[7], Seiji Komeda[5,], Yoshihisa Hirota[1,2,+,*]*

[1]Laboratory of Biochemistry, Department of Bioscience and Engineering, College of Systems Engineering and Science, Shibaura Institute of Technology, Saitama, Saitama 337-8570, Japan.

[2]Medicinal Chemistry and Organic Synthesis, Department of Systems Engineering and Science, Graduate School of Engineering and Science, Shibaura Institute of Technology, Saitama, Saitama 337-8570, Japan.

[3]Department of Urology, Jichi Medical University Saitama Medical Center, Saitama, Saitama 330-8503, Japan.

[4]Division of Hematology and Oncology, Department of Internal Medicine, University of Cincinnati College of Medicine, Cincinnati, OH, 45267, USA.

[5]Faculty of Pharmaceutical Sciences, Suzuka University of Medical Science, Suzuka, Mie 513-8670, Japan.

[6]Laboratory of Organic Synthesis and Medicinal Chemistry, Department of Bioscience and Engineering, College of Systems Engineering and Science, Shibaura Institute of Technology, Saitama, Saitama 337-8570, Japan.

[7]Department of Pharmaceutical Organic Chemistry, Faculty of Pharmacy, Osaka Medical and Pharmaceutical University, Takatsuki, Osaka 569-1094, Japan.

[+]These authors contributed equally to this work.

E-mail address: hirotay@shibaura-it.ac.jp (Y. Hirota) and komedas@suzuka-u.ac.jp (S. Komeda).

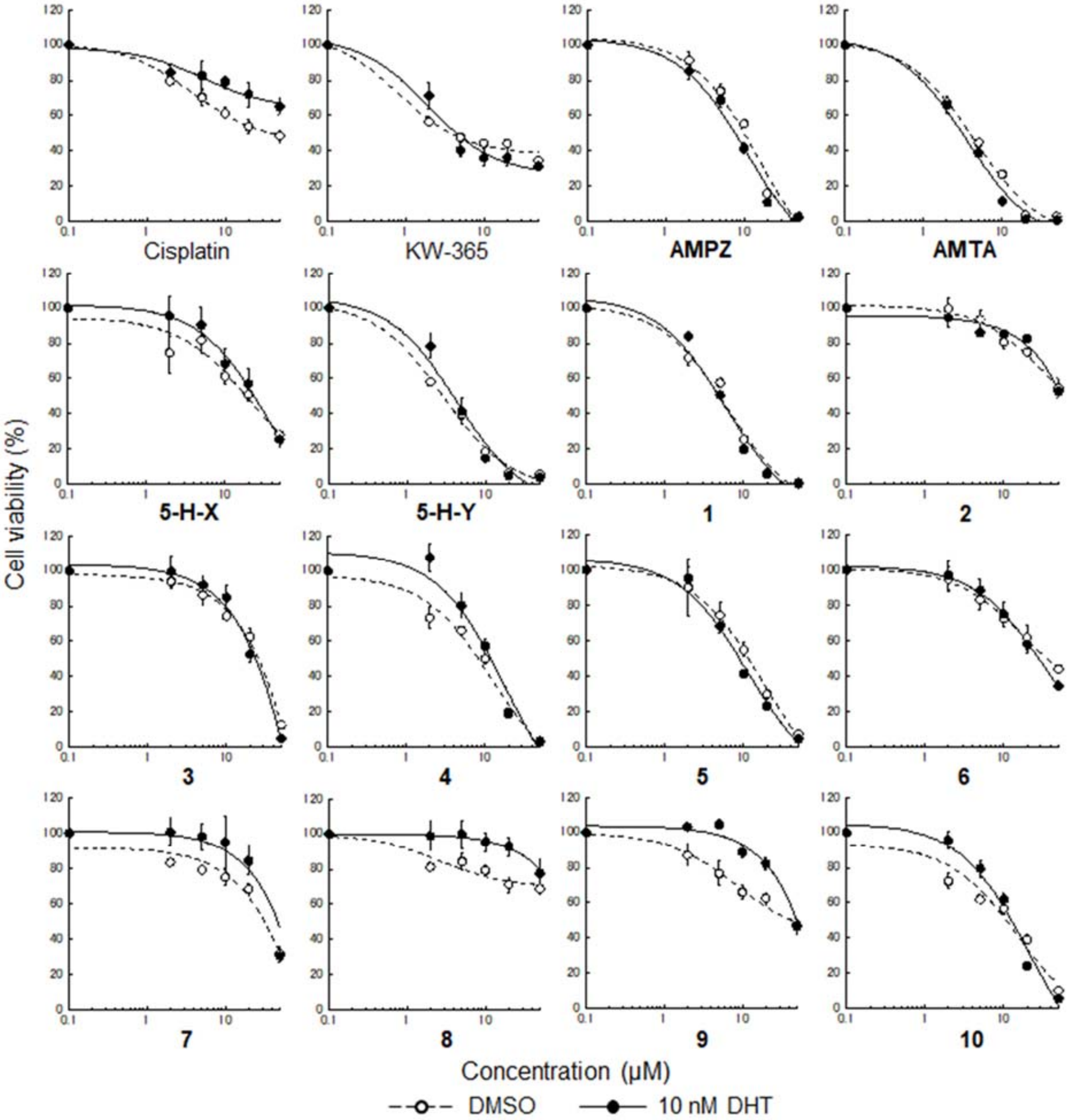

Figure S1. Inhibitory effect of cisplatin, KW-365 and the series of azolato-bridged dinuclear platinum(II) complexes (**AMPZ**, **AMTA**, **5-H-X**, **5-H-Y**, and **complexes 1-10**) on cell proliferation of AR-positive prostate cancer cell line LNCaP by stimulation with DHT.
